# Integrin α11β1 is a receptor for collagen XIII

**DOI:** 10.1007/s00441-020-03300-y

**Published:** 2020-12-11

**Authors:** Jarkko Koivunen, Hongmin Tu, Antti Kemppainen, Padmanabhan Anbazhagan, Mikko A. Finnilä, Simo Saarakkala, Jarmo Käpylä, Ning Lu, Anne Heikkinen, André H. Juffer, Jyrki Heino, Donald Gullberg, Taina Pihlajaniemi

**Affiliations:** 1 Oulu Center for Cell-Matrix Research, Faculty of Biochemistry and Molecular Medicine, University of Oulu, P.O. Box 5400, FIN-90014 Oulu, Finland; 2 Biocenter Oulu and Faculty of Biochemistry and Molecular Medicine, University of Oulu, P.O. Box 5000, FIN-90014 Oulu, Finland; 3 Research Unit of Medical Imaging, Physics and Technology, University of Oulu, P.O. Box 5000, FIN-90014 Oulu, Finland; 4 Department of Biochemistry and MediCity Research Laboratory, University of Turku, Tykistökatu 6A, 20520 Turku, Finland; 5 Department of Biomedicine and Center of Cancer Biomarkers, University of Bergen, Jonas Lies vei 91, N-5009 Bergen, Norway

**Keywords:** Collagen, Integrin, Cell adhesion, Bone homeostasis, ECM receptors

## Abstract

Collagen XIII is a conserved transmembrane collagen mainly expressed in mesenchymal tissues. Previously, we have shown that collagen XIII modulates tissue development and homeostasis. Integrins are a family of receptors that mediate signals from the environment into the cells and vice versa. Integrin α11β1 is a collagen receptor known to recognize the GFOGER (O=hydroxyproline) sequence in collagens. Interestingly, collagen XIII and integrin α11β1 both have a role in the regulation of bone homeostasis. To study whether α11β1 is a receptor for collagen XIII, we utilized C2C12 cells transfected to express α11β1 as their only collagen receptor. The interaction between collagen XIII and integrin α11β1 was also confirmed by surface plasmon resonance and pull-down assays. We discovered that integrin α11β1 mediates cell adhesion to two collagenous motifs, namely GPKGER and GF(S)QGEK, that were shown to act as the recognition sites for the integrin α11-I domain. Furthermore, we studied the in vivo significance of the α11β1-collagen XIII interaction by crossbreeding α11 null mice (*Itga11*^*−/−*^) with mice overexpressing *Col13a1* (*Col13a1*^*oe*^). When we evaluated the bone morphology by microcomputed tomography, *Col13a1*^*oe*^ mice had a drastic bone overgrowth followed by severe osteoporosis, whereas the double mutant mouse line showed a much milder bone phenotype. To conclude, our data identifies integrin α11β1 as a new collagen XIII receptor and demonstrates that this ligand-receptor pair has a role in the maintenance of bone homeostasis.

## Introduction

The adhesion of cells to the neighboring cells and to the extracellular matrix (ECM) is critical for their survival, migration, differentiation, and signal transduction. Integrins are an important family of molecules involved in cell-ECM adhesion process. They are heterodimeric membrane glycoproteins consisting of non-covalently associated α and β subunits (Pandolfi et al. 2017; Barczyk et al. 2010). The ligand-binding specificity of most integrins is well known (Pandolfi et al. 2017; Barczyk et al. 2010). Four collagen-binding integrins, α1β1, α2β1, α10β1, and α11β1, are expressed in various tissues (Hemler et al. 1986; Camper et al. 1998; Gullberg et al. 1995). The collagen receptor integrin α subunits are characterized by an inserted domain named as the I domain (often called as the A domain) that mediates interaction with native collagens (Hamaia and Farndale 2014; Zeltz and Gullberg 2016).

Collagen XIII is a type II transmembrane protein composed of a short cytosolic head, a transmembrane anchor, and a long extracellular domain with three collagenous domains (COL1-3) and four non-collagenous domains (NC1–4) (Hägg et al. 1998; Heikkinen et al. 2012). Our previous in vitro studies using solid-phase assays showed that the I domain of the integrin α1 subunit binds to collagen XIII with similar approximated avidity as to collagen IV (Nykvist et al. 2000), but the binding site in the collagen was not characterized. In another report, we suggested that collagen XIII mediates integrin α1β1-dependent transmigration of monocytes in renal fibrosis with a binding site different from other integrin I domain binding motifs (Dennis et al. 2010). These data prompted us to investigate the binding of collagen XIII to other collagen receptor integrins.

Collagen XIII is broadly distributed in mesenchymal tissues at low levels (Hägg et al. 2001; Sund et al. 2001; Sandberg et al. 1989; Juvonen et al. 1992; Sandberg-Lall et al. 2000). Many studies with genetically modified mice have indicated its physiological functions in musculoskeletal tissues (Ylönen et al. 2005; Härönen et al. 2017, 2019; Zainul et al. 2018; Koivunen et al. 2019) and congenital myasthenic syndrome type 19 (CMS19; OMIM #616,720) musculoskeletal phenotypes (Logan et al. 2015; Rodriguez Cruz et al. 2019).

Integrin α11β1 is a collagen receptor (Lu et al. 2010) recognizing the GFOGER (O=hydroxyproline) sequence in interstitial collagens (W. M. Zhang et al. 2003). We consider of interest that both collagen XIII and integrin α11β1 are induced in several tumor tissues (Väisänen et al. 2005, H. Zhang et al. 2018, Izzi et al. 2019, Mirtti et al. 2006, Zhu et al. 2007, Primac et al. 2019) and that they are involved in the regulation of bone development and homeostasis (Blumbach et al. 2012; Shen et al. 2019; Ylönen et al. 2005; Koivunen et al. 2019). Therefore, we chose to analyze the interaction between these two molecules. We found that α11β1 mediates cell spreading on a collagen XIII-coated surface and that two collagenous motifs, GPKGER and GF(S)QGEK, in the collagen XIII ectodomain contribute in the in vitro binding to the integrin α11-I domain. Additionally, we show that collagen XIII-integrin α11 interaction participates in the maintenance of bone homeostasis in vivo.

## Materials and methods

### Cells

Murine C2C12 myoblast cells from the American Type Culture Collection (ATCC) were stably transfected with a human integrin α2 cDNA or a human integrin α11 cDNA (Tiger et al. 2001), the cDNA encoding the integrin α11 was tagged with enhanced green fluorescent protein (EGFP) C-terminally (Erusappan et al. 2019). Mouse embryonic fibroblast cells (MEFs) were isolated from wild-type mouse embryos as described previously (Popova et al. 2004, 2007) and cultured at 37 °C in DMEM (Biochrom AG, Berlin, Germany) supplemented with 10% FCS (Euroclone, UK) and antibiotics (Cambrex, USA). Immortalized cell lines were created by induction of simian virus 40 (SV40) as described previously (Barczyk et al. 2013).

### Production of integrin I domains

The recombinant I domains of the human integrins α1 and α2 were produced as described previously (Nykvist et al. 2000). Briefly, the α1-I domain cDNA was generated by PCR using a human integrin α1 cDNA (Briesewitz et al. 1993) as a template, cloned into the pGEX-4T-3 vector with a GST tag (GE Healthcare, USA), and the protein was expressed in *E. coli*. The α2-I domain cDNA was produced by PCR using a human integrin α2 cDNA (Takada and Hemler 1989) as a template, cloned into the pGEX-2T vector (GE Healthcare, USA) and the GST- α2I fusion protein was expressed in *E. coli*. The α11-I domain cDNA was generated similarly by PCR using a human integrin α11 cDNA (Velling et al. 1999) as a template, and then cloned into the pAcSecG2T vector (Invitrogen, USA). The α11-I domain was expressed as a GST-tagged fusion protein and secreted into cell culture media in insect High-Five cells (Invitrogen) using a Baculovirus expression system (Invitrogen). All recombinant GST-I domains were purified using glutathione-Sepharose affinity chromatography (GE Healthcare) and their purities were analyzed by SDS-PAGE.

### Production of the recombinant human collagen XIII ectodomain, its collagenous peptides and triple-helical motifs

The ectodomain of human collagen XIII was produced as described previously (Tu et al. 2002). High Five insect cells (Invitrogen) were cultured in a monolayer and co-infected with a virus encoding the human collagen XIII α1 chain lacking the cytosolic domain (Snellman et al. 2000) and another virus encoding both the α and β subunits of human prolyl 4-hydroxylase (Lamberg et al. 1996; Nokelainen et al. 1998). The ectodomain was purified from ~ 400 ml of 48-h infected culture media sequentially using a HiTrap™ Q 5 ml column (GE Healthcare), a HiTrap™ SP 5 ml column (GE Healthcare), and a HiLoad™ Superdex™ 200 column (GE Healthcare). Its purity was analyzed by native gel electrophoresis and SDS-PAGE.

The collagenous peptides of collagen XIII were produced by enzymatic digestion of 400 μg of the collagen XIII ectodomain with 0.4 μg of pepsin at pH 2–3 for 30 min at room temperature and then isolated by a Superdex-200 column in 20 mM HEPES buffer, pH 7.0, containing 0.3 M NaCl.

The recombinant proteins composing the testing motifs were produced in E. coli or insect cells. The DNA fragment encoding (GPP)_5_GFPGER(GPP)_5_ was produced by a two-step PCR reaction. In the first step, the template for the reaction 1 was formed from annealing of two synthetic oligonucleotides 5′-GCCATGGCTGGACCCCCCGGCCCTCCTGGACCTCCTGGTCCCCCTGGTCC-3′ and 5′-TGGTCCCGGTGGACC*ACGCTCGCCAGGGAAACC*GGGAGGACCAGGGGGACC-3′. The only primer 5′-ACAAGGCCATGGCTGGACCCCCC-3′ was used for forward extension. The template for the reaction 2 was from annealing of another pair of oligos 5′-ACCGGATCCCGGTGGGCCAGGTGGTCCGGGTGGACCCGGTGGTCCCGGTGGACC-3′ and 5′-GGTCCCCCTGGTCCTCCC**GGTTTCCCTGGCGAGCGT**GGTCCACCGGGACCA-3′ and a primer 5′-GTAACCGGATCCCGGTGGGCCAGGT-3′ for the reverse extension. After five cycles, the two reaction products were mixed and 25 more PCR cycles continued.

The cloning of other motifs followed the same strategy except changes in two oligonucleotides for annealing, one in italic and underlined, and another in bold and underlined.

The final PCR products were digested with NcoI and BamHI, and the purified DNA was inserted into a plasmid pHisTRX_2_-Foldon (a gift kindly from J. Engel). The DNA sequence was confirmed by DNA sequencing. The DNAs encoding (GPP)_5_motif(GPP)_5_-foldon were further cloned by PCR using primers containing KpnI and HindIII cleavage sites (forward primer AGCTC*GGTACC*AGGACCCCCCGGCCCT; reverse primer CTAATTAAGCTTTTACAGGAAGGTAGA), and the enzymatic digested PCR products were inserted into a plasmid pQE3.1 (Qiagen) for expressing the recombinant protein composing 6xHis-(GPP)_5_motif(GPP)_5_-foldon in an *E. coli* strain BL21(DE3). To express the fusion proteins in insect cells, the DNA fragments were cloned by PCR using a forward primer TCTGCA*GCGGCCGC*ATGAGAGGATCTCACCAT and a reverse primer CTTCTAGAATTCTTATTACAGGAAGGTAGA and then inserted to the expression vector pVL1392 (Invitrogen) through the NotI and EcoRI cleavage. The recombinant fusion proteins were produced from the cell lysates by His-tag affinity purification using Ni-NTA resin (Qiagen). The peptide trimerization was confirmed by a gel filtration chromatography.

### Cell attachment assay

The cells attached to the culture plates were monitored directly by microscopy or quantitatively analyzed using a β-hexosaminidase release assay (Landegren 1984). The ligands used for the cell attachment assays were human plasma fibronectin (Invitrogen), rat tail collagen I (Collaborative Biomedical Products, USA), collagen XIII ectodomain, and its motif-foldon fusion proteins, at a concentration of 10 μg/ml in PBS or in 20 mM HEPES, 0.15 M NaCl, pH 7.0, 0.2% heat-inactivated BSA (crystallized, Serva, Germany) in PBS was used as a blank control. The 24-well cell culture plates (Greiner Bio-one, Germany) were coated with 300 μl of the ligands at 4 °C overnight. The plates were rinsed 3 times in PBS, and the uncoated surface was then blocked with 0.2% heat-inactivated BSA in PBS at 37 °C for 1 h and washed with Puck’s saline (137 mM NaCl, 5 mM KCl, 4 mM NaHCO_3_, 5.5 mM D-glucose, and 2 mg/L phenol red equilibrated with 5% CO_2_ at 37 °C) containing 2 mM MgCl_2_ and 20 µM CaCl_2_. For the attachment testing, C2C12 cells at 60–80% confluence were detached by trypsin-EDTA treatment and washed once with DMEM containing FCS to inactivate the trypsin and then 3 times with Puck’s saline. The cells were added to the wells at a density of 100,000 cells/0.5 ml/well. They were allowed to attach for 1 h at 37 °C in Puck’s saline, and non-attached cells were removed by washing for 3 times with Puck’s saline. For the β-hexosaminidase assay, the attached cells were lysed for 2 h at 37 °C in 200 μl 0.05 M citrate, pH 5.0, containing 0.25% Triton X-100 and 3.75 mM p-nitrophenyl-N-acetyl-β-D-glucosaminide (Sigma, USA). Fifty microliters of the cell lysates were loaded onto a 96-well microtiter plate (Nunc, Denmark) and the reaction was stopped, and color developed by adding 75 μl of 45 mM glycine solution, pH 10.4, containing 4.5 mM EDTA. The absorbance at 405 nm was measured using a Victor microplate reader (Perkin Elmer, USA). A cell number calibration curve was made by incubating known numbers of cells under equivalent conditions. Three repeated experiments were performed in the tests. The data were normalized by taking adhesion to a fibronectin-coated surface as 100% cell attachment and that to wells coated with BSA only as 0%. In the inhibition assay, motif-fusion proteins, with the molar ratios of 1:1, 5:1, and 10:1 to the coating collagen XIII, were mixed with the cells first and then added to the collagen XIII-coated plate wells.

### Immunofluorescence staining and EGFP imaging

The glass coverslips were coated and blocked in the same way as for the 24-well plates in the cell attachment assay. The cells were allowed to adhere at a density of 9000 cells/30 µl/coverslip in DMEM containing 10% FCS or in serum-free DMEM for 1.5–2 h at 37 °C complemented with 5% CO_2_. After washing the coverslips with PBS, the attached cells were fixed in acetone at − 20 °C for 8 min or in 4% PFA at room temperature for 10 min and then permeabilized in acetone at − 20 °C for 5 min. Non-specific binding sites were blocked by incubating with 10% goat serum (Zymed Laboratories, Inc., USA) diluted in PBS. The fixed cells were incubated with the antibody against integrin α11 cytoplasmic domain (Popova et al. 2004) for 1–2 h at 37 °C or at 4 °C overnight. Cy2-, Cy3- (Jackson ImmunoResearch Laboratories, USA), Alexa Fluor 488-, and Alexa Fluor 568-conjugated secondary antibodies (Invitrogen) were used for detection. The stained cells were mounted in Immu-Mount mounting solution (Thermo Shandon, USA), visualized and photographed under a Leitz Aristoplan microscope (Leica Microsystems, Germany) or an Olympus BX51 (Olympus, Japan) equipped with optics for observing fluorescence. To visualize C2C12-α11 + , cells were fixed in 2% PFA + 0.1% Triton X-100 and mounted as described above.

### Western blotting

To verify the expression of integrin α11, C2C12 cells in one culture plate (Φ = 100 mm) at 80–90% confluence were washed once with 10 ml of PBS and then detached with a cell scraper into 1 ml of PBS. The cells were harvested by centrifuging at 340×*g* for 10 min and then homogenized for 1 min with 0.5 ml of 70 mM Tris/0.3 M NaCl/0.2% Triton X-100, pH 7.4, containing Protease Inhibitor cocktail (Roche Applied Science, Germany), followed by incubation on ice for 30 min. After centrifuging at 8000×*g* for 10 min, the supernatant of the cell lysate was applied to SDS-PAGE followed by western blotting. The expression of recombinant human integrin α11 in C2C12 cells was detected with an affinity-purified antibody against the human α11 cytoplasmic domain (Tiger et al. 2001).

### Solid-phase binding and surface plasmon resonance assays

Fifty microliters of collagen solutions, collagen I (Collaborative Biomedical Products, USA), collagen IV (BD Biosciences), collagen XIII ectodomain, its pepsin-digested fragments, and the recombinant motif-fusion proteins, were coated on the plastic surfaces of MaxiSorp™ 96-well microplates (Nunc) at 4 °C overnight. All the other steps were performed at room temperature using a buffer containing 20 mM Tris, 0.15 M NaCl, 2 mM MgCl_2_, 20 µM CaCl_2_, 0.05% Tween-20, or 10 mM HEPES, 0.15 M NaCl, 2 mM MgCl_2_, 20 µM CaCl2, pH 7.0. Three parallel samples were analyzed. The wells were blocked with 5% fat-free milk in Tris buffer or in HEPES buffer for 1 h and the samples incubated for 1.5 h with α1-, α2-, and α11-I domains diluted in the buffer containing 30 μg/ml BSA (crystallized, Serva). After thorough washing, the binding ligands were detected with a goat polyclonal anti-GST antibody (Rockland Immunochemicals, USA) and then with an anti-goat IgG secondary antibody conjugated with horseradish peroxidase (Dako, Denmark). TMB-Xtra (Kem-En-Tec Diagnostics A/S, Denmark) was used as the substrate for detection.

Surface plasmon resonance (SPR) analysis was performed at 25 °C in a BIAcore® 3000 system (Biacore AB, Sweden) using a CM5 sensor chip (Biacore). Integrin α11-I domain was immobilized onto the chip at a level of 2000 resonance units (RUs). Binding of collagen XIII was tested in 10 mM HEPES, pH 7.0, 0.15 M NaCl, 2 mM MgCl_2_, 20 μM CaCl_2_ containing 0.005% P-20 surfactant (Biacore) at a flow rate of 20 μl/min. The blank control was carried out on one of the flow cells on the sensor chip lacking the immobilized I domain protein.

### Pull-down assay

Insect High-Five cells were infected with viruses encoding GST and GST-α11-I domain respectively with MOI of 10 in TNM-FH media (Sigma) containing 10% FCS. Eighty percent of the recombinant proteins were secreted into the media due to a signaling peptide in the expression vector. Forty-eight postinfection 5 ml media from each cell culture plate were collected and dialyzed in PBS containing 0.1 mM DTT and 0.001% Triton. The media were then mixed with the glutathione-Sepharose pre-equilibrated with the dialysis buffer at 4 °C overnight. After washing out the unbound proteins, the purified collagen XIII ectodomain was incubated with the Sepharose resin in 10 mM HEPES, pH 7.0, containing 0.15 M NaCl and 2 mM MgCl_2_ at room temperature for 2 h. The unbound collagen XIII was removed by extensive washing steps and the proteins absorbed on the glutathione-Sepharose were eluted by incubating with either 10 mM glutathione or one cleavage unit of thrombin at room temperature overnight. The unbound collagen XIII was incubated at room temperature overnight as a control. The elution fractions were analyzed with a western blotting using a monoclonal antibody recognizing the recombinant human collagen XIII (Tu et al. 2002).

### Sequence retrieval and alignment

The full-length human integrin α11 sequence is 1188 residues in length. We retrieved only the I domain residues ranging from 164 to 345 which comprises 182 residues in total (later renumbered from 1 to 182) from the UniProtKB (**Q9UKX5**). The crystal structure of the human integrin α2-I domain bound with the collagen peptide (PDB code: 1DZI) as well as the sequence information was obtained from the PDB (Berman et al. 2000). Sequence alignment between the integrin domains of α2 and α11 was carried out using ClustalW2 (Larkin et al. 2007) with default settings.

### Homology modeling of the human integrin α11-I domain

The final sequence alignment obtained from the CLUSTALW2 program was submitted to the MODELLER9v1 software package (Eswar et al. 2006; Sali and Blundell 1993) for constructing the homology model of the human integrin α11-I domain. Twenty different models were generated and the model with the lowest energy was considered as the ideal model.

### Energy minimization and structure validation

Energy minimization of the human integrin α11-I domain model was carried out using GROMACS version 4.0.7. (Hess et al. 2008; Van Der Spoel et al. 2005). Initially, the protein was placed in a cubic box and a SPC water model was used for solvation. After solvating, suitable ions were added to neutralize the whole system. The final system was composed of 13,073 molecules, which includes the protein (182 residues), water (12,882 molecules), and ions (9 Na^+^). The whole system was then subjected to 1000 steps of steepest descent minimization using the GROMOS96 53a6 force field until the maximum force converged to < 1000.0 kJ/mol/nm with PME (Particle Mesh Ewald) as the Coulomb type, the *r*coulomb cutoff of 1.0 and van der Waals cutoff of 1.0. For validation purposes, the minimized final model was submitted to various structure validation programs (PROCHECK, VERIFY3D and ERRAT) available through SAVeS server (https://servicesn.mbi.ucla.edu/SAVES/).

### Collagen triple-helical peptide construction

The crystal structure of the α2-I domain-collagen peptide complex was downloaded from the PDB databank, and then collagen triple-helical peptides (strands B, C, D) were manually separated from the integrin domain. Each peptide sequence comprises of 21 amino acid residues (the residues in each peptide strands were later renumbered from 1 to 21). Here, we constructed three different collagen triple-helical peptides namely, (GPP)_2_GFQGEK(GPP)_3_, and (GPP)_2_GPKGER(GPP)_3_. These collagen triple-helical peptides were constructed using COOT software package (P. Emsley et al. 2010). All these constructed triple-helical peptides were then subjected to 500 steps of steepest descent minimization using GROMACS version 4.0.7 (Hess et al. 2008; Van Der Spoel et al. 2005). The final energy minimized triple-helical peptides were then used for docking with the human integrin α11-I domain.

### Protein-protein docking

Docking of modeled human α11-I domain and the constructed triple-helical peptides (α11-(GPP)_2_GFQGEK(GPP)_3_ and α11-(GPP)_2_GPKGER(GPP)_3_ were carried out using the HADDOCK program (Dominguez et al. 2003), a Web-based tool to perform protein-protein docking. The HADDOCK docking program is driven based on the Ambiguous Interaction Restraints derived from the experimental information of the intermolecular interacting residues provided by the user. We used the easy interface for all the docking calculations. However, before carrying out the docking experiments between the modeled human α11-I domain and collagen XIII peptides, we first performed a control docking experiment using the crystal structure of ligand-binding α2-I domain to validate the HADDOCK docking program. Upon docking of α2-I domain and its collagen triple-helical peptide (GFOGER), the program had predicted the key interacting residues as seen in the crystal structure suggesting that HADDOCK can be used for the docking calculations using the triple-helical peptides. The constructed peptides were then docked to the modeled α11-I domain. The best representative docked complex was chosen based on the lowest energy cluster and HADDOCK score obtained through diverse energy terms such as van der Waals energy, electrostatic energy, desolvation energy, restraints violation energy and buried surface area. PDBsum database was used to analyze the protein-peptide interactions while the binding energy (ΔG) was calculated using the PRODIGY server (Xue et al. 2016).

### Mouse line

Generation of the transgenic *Col13a1*^*oe*^ mice has been described previously (Ylönen et al. 2005), and the mouse line is publicly available in the Infrafrontier EMMA repository with the strain name B6.Cg-Tg(Col13a1)2Pih/Oulu (EM:09885). Generation of *Itga11*^*−/−*^ mice has been described previously (Popova et al. 2007). Here, *Itga11*^*−/−*^ mice in C57BL/6J background were bred with *Col13a1*^*oe*^ to generate a *Col13a1*^*oe*^*;Itga11*^*−/−*^ double mutant mouse line, overexpressing *Col13a1* and lacking *Itga11*. *Col13a1*^*oe*^ littermates were used as controls. Permission for the maintenance of mice (license ID ESAVI/4220/04.10.07/2013) was obtained from the Finnish Animal Care and Use Committee of the State Provincial Office of Southern Finland (Hämeenlinna, Finland) and the European Community Council Directive on the protection of animals used for scientific purposes (September 22, 2010; 2010/63/EEC), national legislation and the regulations for the care and use of laboratory animals were followed.

### µCT

Tubular bone microarchitecture was assessed from left femurs of *Col13a1*^*oe*^;*Itga11*^*−/−*^ mice, and they were compared to *Col13a1*^*oe*^ mice. Number of mice analyzed in order of increasing age: female *Col13a1*^*oe*^ (7, 4, 6, 6, 6), female *Col13a1*^*oe*^;*Itga11*^*−/−*^ (3, 3, 4, 3, 2), male *Col13a1*^*oe*^ (5, 4, 7, 6), male *Col13a1*^*oe*^;*Itga11*^*−/−*^ (3, 3, 3, 3). The most recent guidelines for bone microstructure assessment were used (Bouxsein et al. 2010). Bones were imaged using a Skyscan 1176 scanner (Bruker microCT, Kontich, Belgium) using the following parameters: X-ray tube voltage of 50 kV, current of 500 µA, exposure time of 4000 ms, and a 0.5 mm aluminum filter. Projection images were collected every 0.3° over a 360° rotation. Image processing was performed with the manufacturer’s software NRecon (v. 1.6.5.2), Dataviewer (v. 1.5.6.2), and CTAn (v. 1.11.10.0). Femur length was measured from reconstructed datasets from the superior top of trochanter major to the distal patellofemoral groove in mid-frontal plane. The average value of three separate measurements was used as the result for the femur length. Measurements were conducted using the manufacturer’s software (Skyscan, Dataviewer v. 1.5.6.2). Besides the femur length, cortical bone morphology was assessed. Diaphyseal cortical bone was assessed using the VOI starting 0.9 mm distally from the femoral neck and extending further distally. Two different ROI lengths were used for cortical bone analysis: 3.6 mm was used for 4-week time point, whereas 5.4 mm was used for other time points. 2D structural analysis was performed to define cortical bone area (Ct.Ar) and average cortical thickness (Ct.Th). 2D volumetric analyses of datasets were conducted using CTAn software (Skyscan, v. 1.11.10.0).

### Statistics

Statistical significance was determined by two-way ANOVA followed by the Benjamini, Krieger, and Yekutieli FDR post hoc test (Benjamini et al. 2006). Values of FDR-corrected *p* (*q*) < 0.05 values were considered statistically significant. No statistical methods were used to predetermine sample size.

## Results

### Integrin α11β1 is localized at focal contacts in cells spreading on a collagen XIII-coated surface

**Fig. 1:**
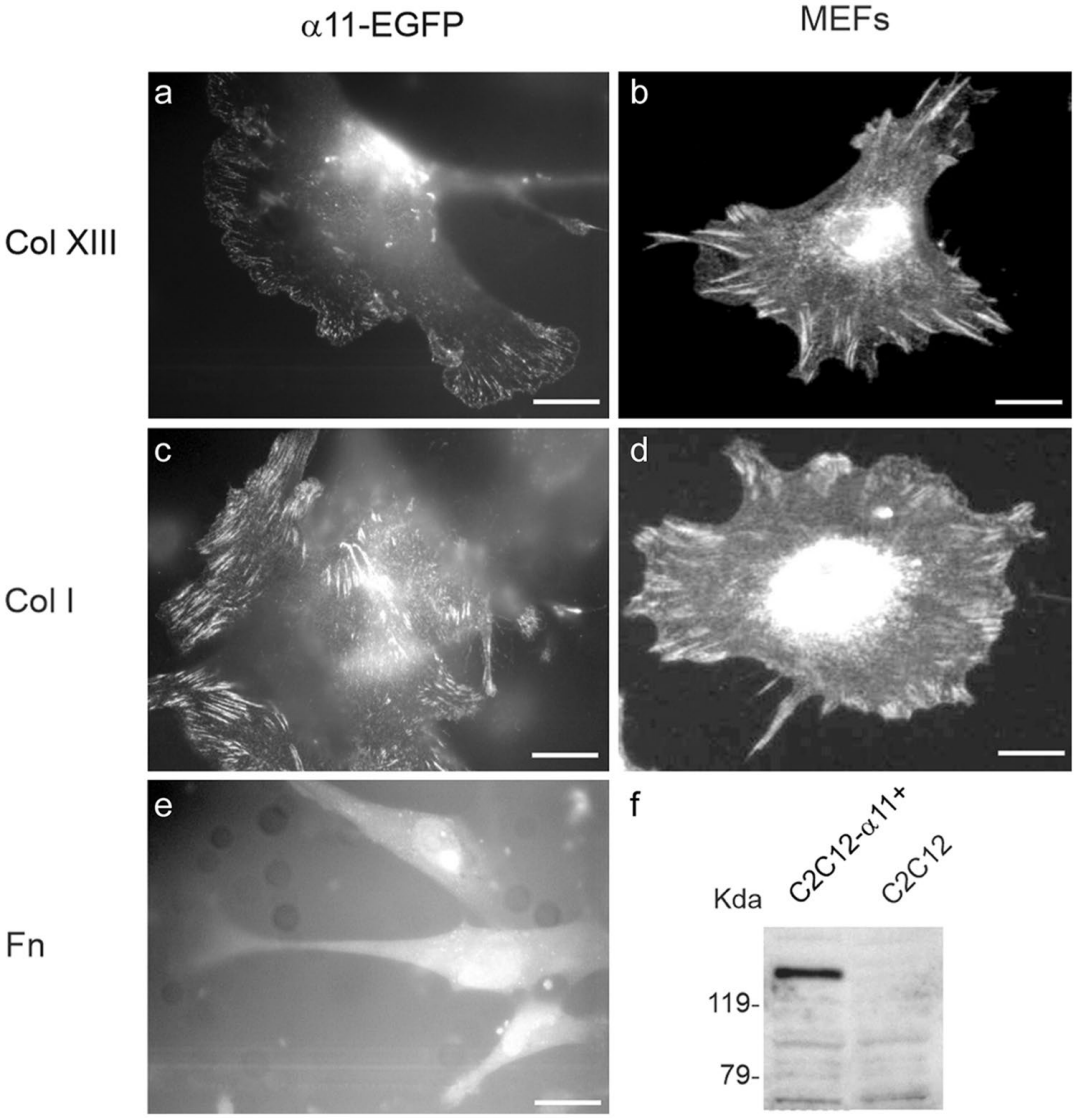
Localization of integrin α11β1 in cells spreading on collagens XIII and I. C2C12 cells transfected with integrin α11-EGFP were seeded onto coverslips coated with collagen XIII (Col XIII) ectodomain **a** and collagen I (Col I) **c** and allowed to adhere at 37 °C for 2 h. The cells were then fixed and imaged on a fluorescence microscope with direct detection of EGFP-tagged protein. **e** The C2C12-α11+ cells spreading on the fibronectin-coated surface. Primary wild type MEFs spread on collagen XIII **b** and on collagen I **d** under the same conditions as the C2C12-α11+ cells and were visualized with indirect immune fluorescence staining using an antibody against the cytoplasmic domain of mouse integrin α11. **e** C2C12 cells were stably transfected with integrin α11, and the expression of α11 was analyzed by western blotting using an antibody against the α11 cytoplasmic domain

To determine whether α11β1 mediates the spreading of cells on collagen XIII, α11-EGFP transfected C2C12 mouse myoblasts (C2C12-α11 +), and mouse embryonic fibroblasts (MEFs) derived from a wild-type mouse embryo were tested. Direct fluorescence imaging of C2C12-α11+ cells showed that α11-EGFP localized at cell-matrix adhesion structures during cell spreading on collagen XIII (Fig. 1a) and on collagen I (Fig. 1c), but not on fibronectin (Fig. 1e). The cellular localization of integrin α11β1 on primary MEFs on collagen XIII was studied by immunofluorescence staining using an antibody against the cytoplasmic domain of the mouse integrin α11 subunit (Fig. 1b). Spreading on collagen I was analyzed as a control (Fig. 1d). These data indicate that both collagen XIII and collagen I, but not fibronectin, are functional ligands for integrin α11β1.

### C2C12 cells expressing integrin α11β1 adhere to collagen I and collagen XIII-coated surfaces

**Fig. 2:**
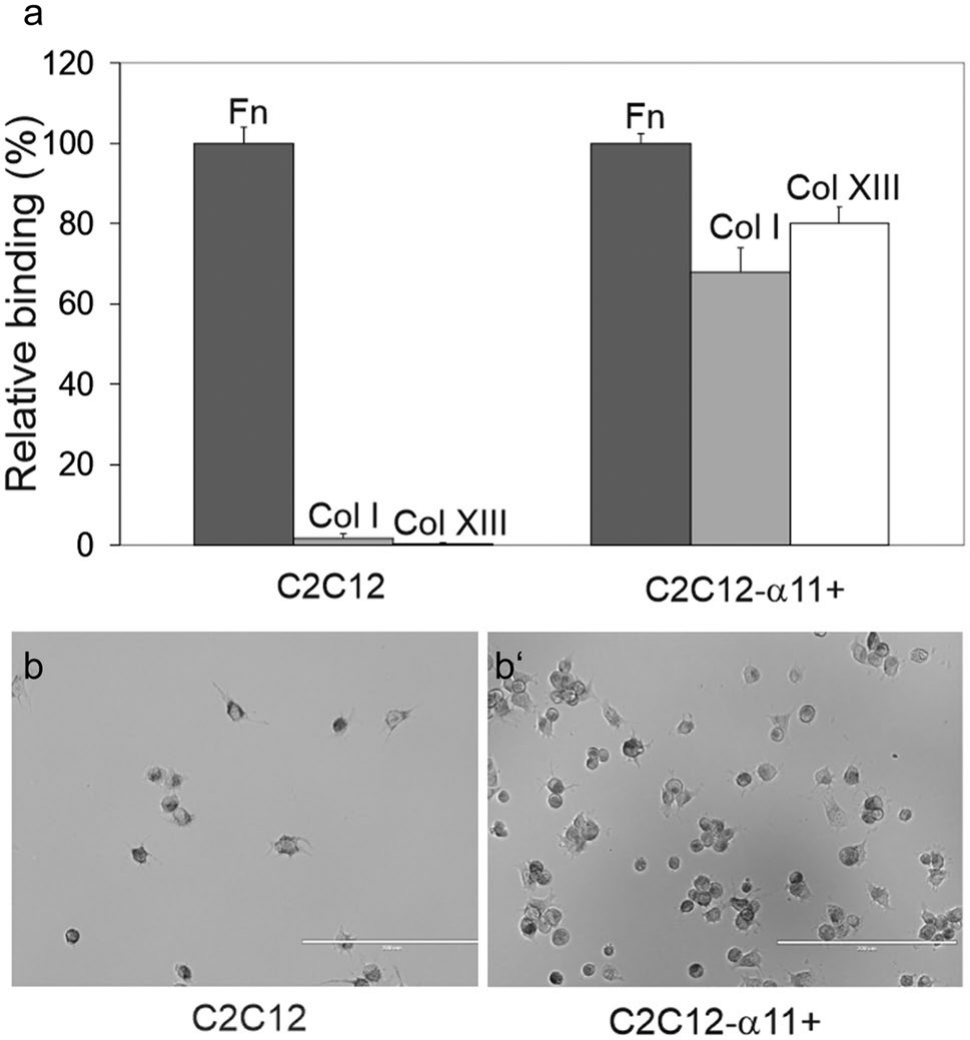
Adhesion of integrin α11-transfected C2C12 cells to fibronectin and collagens (a) C2C12 control cells and cells transfected with an integrin α11cDNA (C2C12-α11+) were seeded on a plastic surface coated with fibronectin, collagen I, or the collagen XIII ectodomain. The cells were analyzed quantitatively using the β-hexosaminidase release assay for the attachment to fibronectin, collagen I, and collagen XIII. Data are presented relative to fibronectin and BSA binding, where the saturated binding to fibronectin represents 100% and the background binding to a BSA-coated surface represents 0%. Values are shown as means of triplicates with standard deviation bars. (b, b’) Microscopic monitoring of the attachment and morphology of C2C12 and C2C12-α11 cells adhering on collagen XIII. Scale bars 200 μm

The interaction between collagen XIII and integrin α11β1 was further studied by using the transfected C2C12 cells that expressed human α11β1 as their only collagen receptor (Tiger et al. 2001). C2C12 cells transfected with the vector plasmid (MOCK) were tested in parallel. The expression of the α11 subunit was verified by western blotting using an antibody specific for the cytoplasmic domain of the human integrin α11 (Fig. 1e). C2C12 control cells did not adhere to native collagen I or to native collagen XIII, but the cells attached well to fibronectin due to the endogenous expression of fibronectin receptor integrins (Fig. 2a). Cell attachment assays utilizing C2C12 cells that were stably transfected with the full-length human integrin α11 cDNA (C2C12-α11+ cells) indicated a remarkable increase in adhesion to collagens I and XIII when compared to control C2C12 cells (Fig. 2a). The attached C2C12-α11+ cells started to spread on the collagen XIII-coated surfaces after 2 h (Fig. 2b). For control purposes, C2C12-α2+ cells were tested with similar results as in our previously published study (Fig. S1), i.e., integrin α2 can mediate cell spreading on collagen I but not on collagen XIII (Nykvist et al. 2000). Integrin α2β1 and α11β1 have both been suggested to be the cellular receptors for fibrillar collagens (Zeltz and Gullberg 2016), but the present results demonstrate a remarkable difference in their preference for collagen XIII.

### Collagen XIII binds to the I domain of integrin α11 subunit with high avidity in vitro

**Fig. 3:**
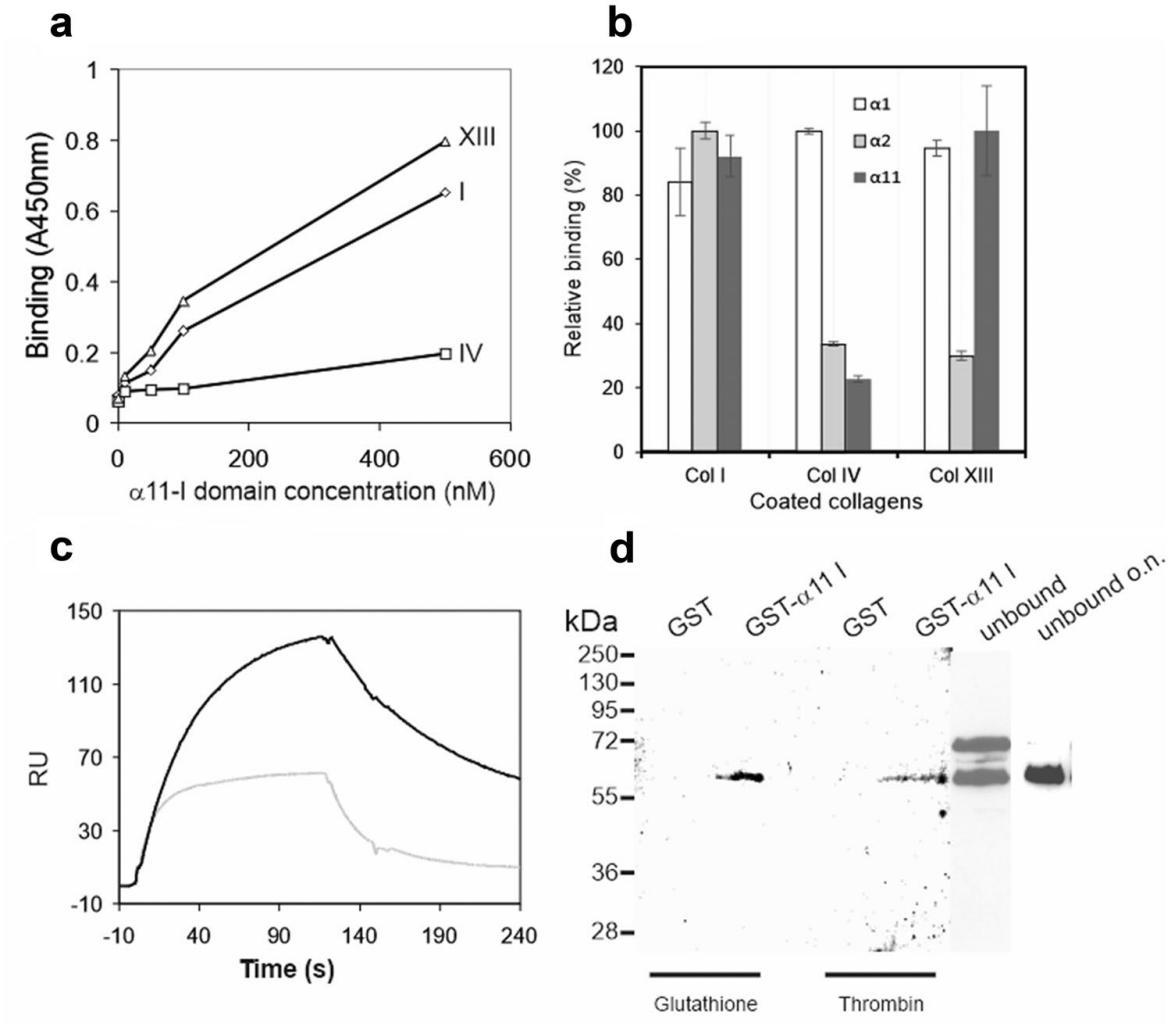
Selective binding of collagen XIII to the integrin α11-I domain; **a** 0.25 μg of collagens I, IV, and XIII in 50 μl of TBS were coated on microtiter plates. The GST-α11-I domain was diluted in series in TBS containing 30 μg/ml BSA, 2 mM MgCl_2_, and 0.05% Tween-20 and used for binding tests employing a solid-phase assay with an antibody against GST. Five percent of fat-free milk in TBS was used for blocking the uncoated surface and for a blank control. **b** A summary of relative binding comparison of I domains to the coated collagen types I, IV, and XIII (Col I, Col IV, and Col XIII, respectively). **c** SPR response of the binding of collagen XIII to the immobilized integrin α11-I domain (black curve). The gray curve shows the background of the sensorgram. **d** A pull-down assay was performed by incubation of collagen XIII with the GST-α11-I domain or GST protein coupled glutathione-Sepharose. The interaction complex was eluted by 10 mM of reduced glutathione or cleaved by thrombin. The eluted, unbound, and unbound incubated overnight (o.n.) collagen XIII was detected by a collagen XIII monoclonal antibody

A GST-α11 I-fusion protein has previously been produced in *E. coli* (W. M. Zhang et al. 2003), but the protein yield and binding activity varied from batch to batch (Käpylä J. et al., unpublished). To facilitate studies on the interaction between integrin α11β1 and collagen XIII in more detail, we set up an expression system of the α11-I domain as a secreted protein in insect cells. The recombinant protein contains a GST-tag and a thrombin cleavage site between GST and the I domain. The α11-I domain was purified from the medium by a one-step GST-affinity purification method. The unpurified media containing GST-tagged α11-I domain or GST as a blank control was used for binding specificity test in vitro. The human collagen XIII ectodomain was expressed as a homotrimer using a baculovirus system in insect cells (Tu et al. 2002). Furthermore, collagen types I and IV were compared to collagen XIII in the binding assays. The integrin α1- and α2-I domains obtained from *E. coli* (Nykvist et al. 2000) which also contained the GST-tags, were included for selected interaction studies. The binding tests were performed using an enzyme-linked immunosorbent assay (ELISA) in which collagens I, IV, and XIII were coated onto the plastic surface as solid ligands and the I domains were used as soluble analytes. The interaction was detected using a GST antibody. The α11-I domain interacted with collagen types I and XIII in a dose-dependent manner (Fig. 3a). Collagen XIII was identified to bind α11-I domain with similar affinity as collagen I and stronger than collagen IV (Fig. 3b). Consistently with the previously published results (W. M. Zhang et al. 2003; Tiger et al. 2001; Nykvist et al. 2000), collagen IV showed preferential binding to the α1-I domain, but no binding to the α11-I domain (Fig. 3b). In contrast to collagens IV and XIII, collagen I bound well to all the tested integrin I domains (Fig. 3b).

The specific interaction between collagen XIII and integrin α11β1 was further confirmed by surface plasmon resonance (SPR) and pull-down assays. In SPR, collagen XIII showed a typical sensorgram of binding to the immobilized fusion protein α11-I domain (Fig. 3c, black curve) unlike in the blank control in which no protein was immobilized (Fig. 3c, gray curve). In a pull-down assay, collagen XIII was trapped only by the GST-α11-I domain coupled to the glutathione-Sepharose but not when GST was used alone. The protein complex could be eluted by glutathione or thrombin, indicating that the interaction site locates in the α11-I domain (Fig. 3d). Due to the long incubation time at room temperature, collagen XIII eluted from the interaction complex was partly degraded, resulting in a reduced molecular mass of 60 kDa. The unbound fraction contained both this 60 kDa fragment as well as the full-length 72 kDa collagen XIII ectodomain, and after overnight incubation, mimicking the enzyme digested fractions, equal degradation of the unbound fraction was evidenced (Fig. 3d).

### Integrin α11-I domain binds specifically to the C-terminal collagenous domain of collagen XIII

**Fig. 4:**
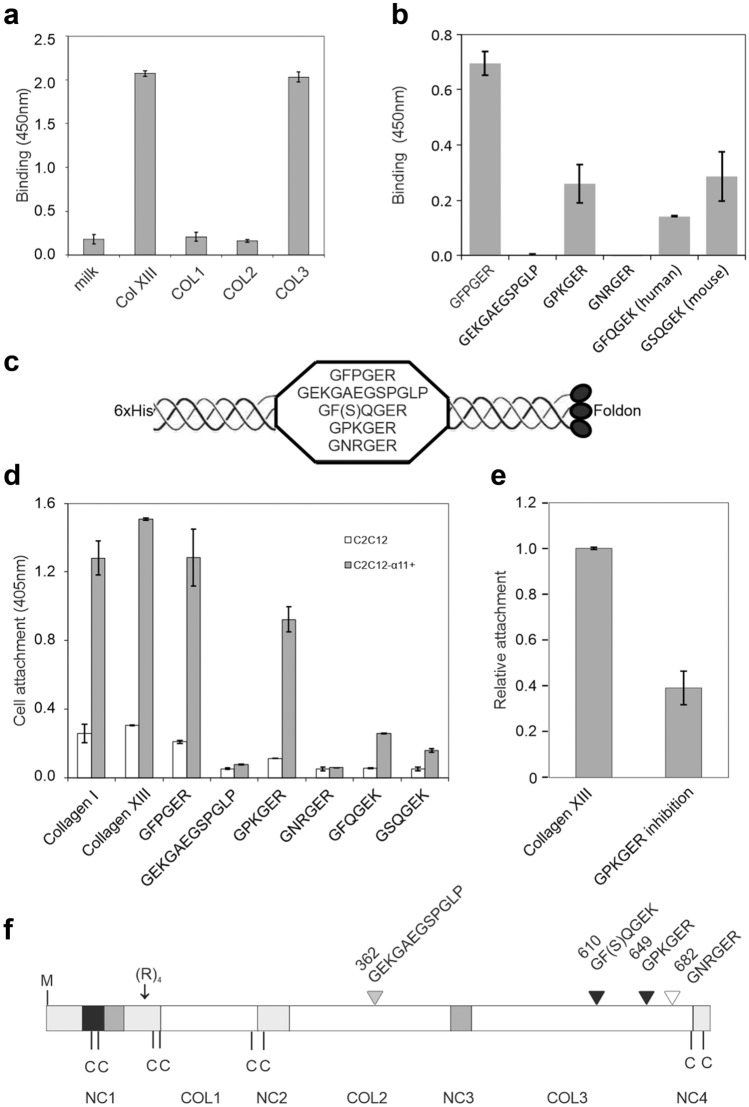
Integrin α11 binds to the COL3 domain of collagen XIII specifically by GPKGER and GF(S)QGEK. **a** A solid-phase binding assay shows binding of the integrin α11-I domain to the COL3 domain specifically. One hundred nanomolars of the collagen XIII ectodomain (Col XIII) and its pepsin-digested collagenous fragments, COL1, COL2, and COL3 in 50 μl of HEPES buffer were coated onto a microtiter plate wells, and 700 nM of the GST-α11-I domain in 50 μl of HEPES buffer containing 2 mM MgCl_2_ was added for binding testing. The bound I domain was detected by a GST antibody. **b** A solid-phase binding assay shows binding of the α11-I domain to the recombinant proteins composed of the testing motifs flanked by the collagenous sequence 5x(GPP) and a foldon sequence at the C-termini. **c**, **d** A cell attachment assay indicates the binding of the specific collagen XIII motifs to the integrin α11. GFPGER was used as positive control. **e** Cell attachment inhibition by the GPKGER motif. **f** A schematic structure of collagen XIII compiled from previous work (Hägg et al. 1998). The black arrowheads indicate the positive binding sites and the white shows a negative binding site. The grey arrowhead points to a reported integrin α1-binding site (Dennis J. et al. 2010). The numbers above the arrowheads represent the residues in the human collagen XIII sequence. The collagenous domains (COL1-3) are indicated as white boxes, the non-collagenous domains (NC1-4) as light gray boxes, coiled-coil motifs as dark gray boxes, and the transmembrane domain as a black box. The initiation methionine is marked as M, cysteine residues as C, and a furin proteolytic cleavage site consisting of four arginine residues as (R)_4_

To identify the potential integrin α11β1 binding sites in collagen XIII, the purified collagen XIII ectodomain was digested by pepsin and the cleaved fragments were separated by gel-filtration chromatography (Tu et al. 2002). The peptide representing the COL3 domain showed similar integrin-binding capability as the intact ectodomain (Fig. 4a). Collagen XIII does not contain the GFOGER or other previously identified integrin-binding motifs (UniProt Consortium 2019). However, the COL3 domain in collagen XIII harbors two GER and seven GEK sequences that are typically found as parts of the known integrin-binding motifs. To test these putative binding sequences, two GER motifs, namely GPKGER and GNRGER, and one GEK motif, namely GF(S)QGEK (“S” in mouse), were expressed in *E. coli* using an expressing vector encoding two (GPP) × 5 sequences flanking the tested fragments, a foldon sequence for triple-helical formation (Frank et al. 2001), and a His-tag for the protein purification (Fig. 4c). GFPGER was chosen as a positive control and GEKGAKGSPGLP, a peptide located on the COL2 domain and binding to integrin α1β1 (Dennis et al. 2010), was produced for a negative control. Other GEK motifs were considered less interesting based on the knowledge gained from the peptide ToolKits of collagen-receptor interactions (Farndale et al. 2008). The motifs GPKGER and GF(S)QGEK showed positive binding to the integrin α11-I domain, albeit at a 50–60% lower approximated avidity when compared to GFPGER (Fig. 4b). GEKGARGSPGLP and GNRGER showed no binding (Fig. 4b). The protein-protein interaction was further confirmed by a cell attachment assay utilizing collagen I, the ectodomain of collagen XIII, and the recombinant proteins containing the putative integrin-binding motifs. The binding of cells was markedly stronger to the GPKGER than to the GF(S)QGEK motif (Fig. 4d). Furthermore, the attachment of C2C12-α11+ cells to the collagen XIII-coated surface could be partially inhibited by GPKGER (Fig. 4e). These data suggested that both GPKGER and GF(S)QGEK contributed to the binding of collagen XIII to the α11-I domain. The two binding sites identified in the COL3 domain of collagen XIII lie within a highly conserved region (Fig. 4f).

### Homology modeling and structure validation

**Fig. 5:**
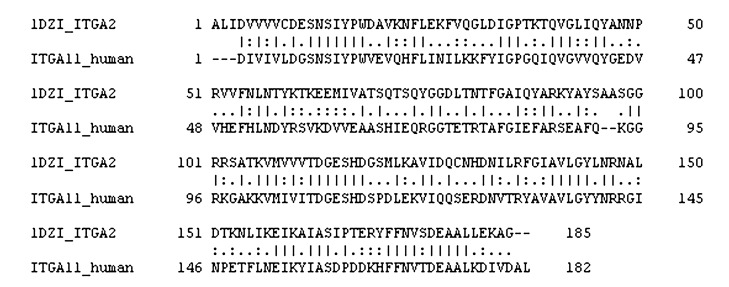
Sequence alignment. Human integrin α2-I (ITGA2) and α11-I (ITGA11) domains share sequence identity of 43.9% and 63.1%, respectively

**Fig. 6:**
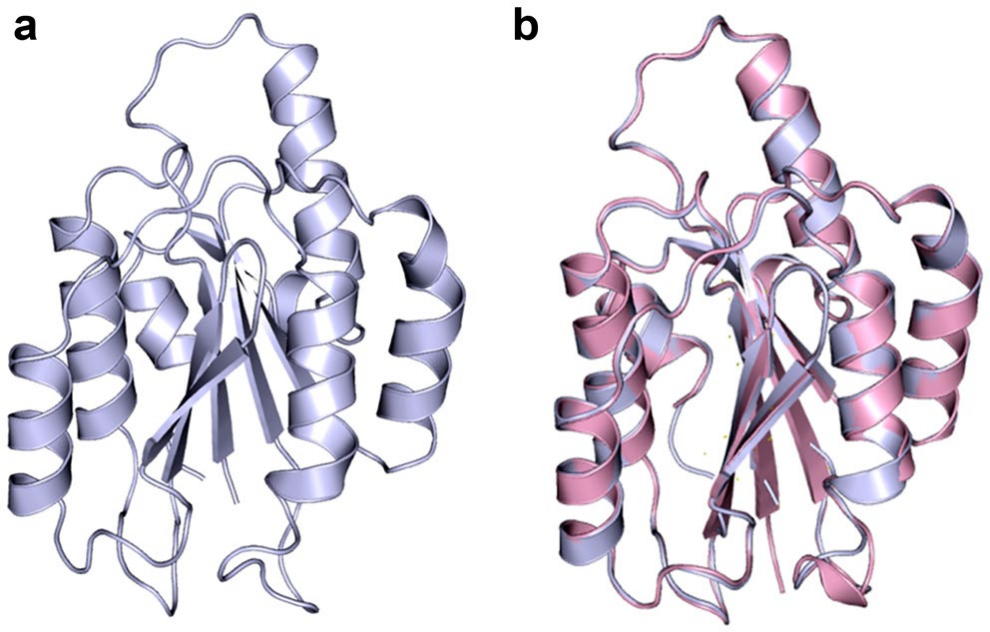
Molecular model of the integrin α11-I domain. **a** An energy minimized structure of human integrin α11-I domain. **b** A superimposed model of the α11-I domain (blue) and the crystal structure of human integrin α2-I domain (pink, PDB: 1DZI) is shown. The root mean square deviation between these two structures is 0.237

To study the molecular interaction between the human α11-I domain and the triple-helical peptides, a homology model of the human α11-I domain was constructed using the crystal structure of the human integrin α2-I domain (PDB code: 1DZI) (J. Emsley et al. 2000) as a template. Prior to the homology model construction, the sequence alignment of α11 and α2 integrin I domains were subjected to manual inspection as a quality check; this identified a shared sequence identity and similarity of 44% and 63.1%, respectively (Fig. 5), being amendable for the further modeling studies. The energy minimized model of the human α11-I domain is shown in Fig. 6a. The root mean square deviation between the final energy minimized model of the α11-I domain and the α2-I domain was 0.237 (Fig. 6b), suggesting that the initial backbone structure was intact and there was not much deviation from its original conformation. Ramachandran plot analysis using PROCHECK (Laskowski et al. 1993) showed that 90.1% of the residues fall in the most favored regions and 9.3% of the residues in the additionally allowed regions (Fig. S2). Also, the assessment of the constructed model by its 3D profile using VERIFY3D (Eisenberg et al. 1997) showed that 100% of the residues are within the allowed regions (Fig. S3). Further, the reliability and the quality of the model were validated using the ERRAT program (Colovos and Yeates 1993). The comparison between the initial model (before minimization) and the final model (after minimization) showed that the quality of the modeled protein was significantly increased after minimization with the overall quality factor score of 97.093 (Fig. S4). The structure validation programs suggest that the constructed homology model of the human α11-I domain structure is of high quality and reliable, thus, can be used for our docking studies.

### Docking of α11-I domain and triple-helical collagen peptides

To study the collagen XIII binding mode to the α11-I domain, we performed docking studies using our homology model of α11-I domain and the HADDOCK program (Dominguez et al. 2003). First, the interface residues that directly interact with the triple-helical collagen were identified from the crystal structure of the α2-I domain. Then, the identified residues were mapped correspondingly to the α11-I domain in the sequence alignment. These residues were then supplied as active residues of the α11-I domain while the passive residues were set to define automatically by the HADDOCK program (Dominguez et al. 2003) to perform the docking calculations.

**Fig. 7:**
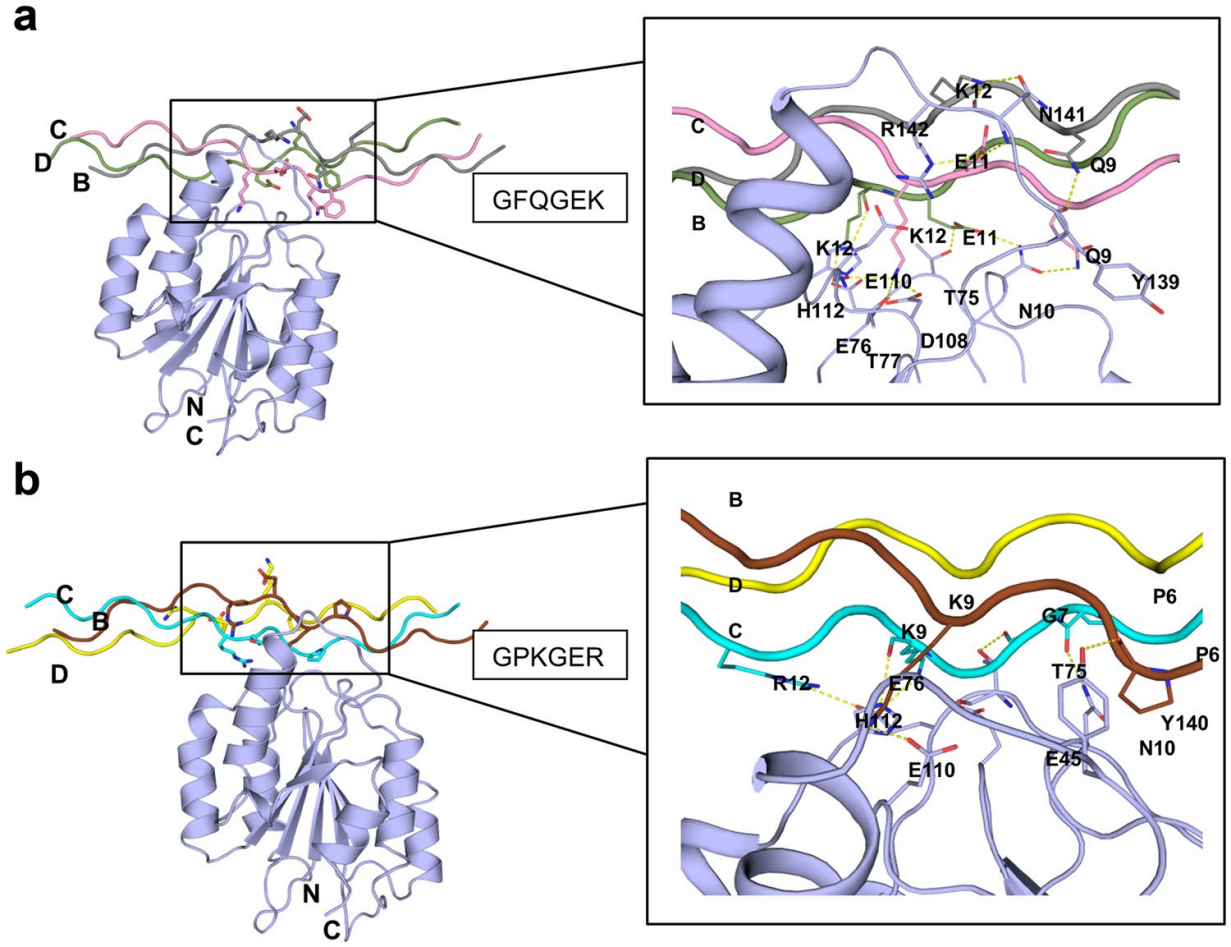
Docked complexes of collagen tripeptides and the α11-I domain. **a** The collagen XIII tripeptide (GPP)_2_GFQGEK(GPP)_3_ bound to the α11-I domain, sidechains of the interacting residues are labelled and shown on the right side. **b** A complex of α11-(GPP)_2_GPKGER(GPP)_3_ along with the interacting residues

The HADDOCK server produced 176 different structures of the α11-(GPP)_2_GFQGEK(GPP)_3_ complex classified in 8 clusters which represents 88.0% of the water-refined models. For the α11-(GPP)_2_GPKGER(GPP)_3_ complex, the HADDOCK server resulted in 136 structures classified in 10 clusters representing 68% of the water-refined models. Analysis of the α11-(GPP)_2_GFQGEK(GPP)_3_ complex revealed that the residues from all the three strands (strands B, C, and D) of the collagen peptide contributed to the interactions with the interface residues of the α11-I domain (Fig. 7a). Lys12 of the strand B, Glu11 and Lys12 of the strand C, and Glu11 and Lys12 of the strand D were involved in the formation of multiple hydrogen bonds with the α11-I domain. Apart from the hydrogen bonds, Glu11 and Lys12 of the strand C, Lys12 of the strand D also participated in the salt bridge formation with the α11-I domain residues. The energy values of the docked complexes are shown in Table 1 and detailed list of hydrogen bonds formed between the atoms of α11-I domain and GFQGEK is shown in Table 2.

**Table 1:** The best representative docked complex was chosen based on the HADDOCK score and the cluster size. Binding energy (**ΔG**) for each of the docked complexes was calculated using the PRODIGY server. Van der Waals, electrostatic energy, and buried surface area obtained from HADDOCK server are shown

Docked Complex	HADDOCK score	Binding energy ΔG (kcal/mol)	Van der Waals energy (kcal/mol)	Electrostatic energy (kcal/mol)	Buried Surface (Å)
α11-I domain-(GPP)_2_GFQGEK(GPP)_3_	− 105.99 ± 9.9	− 6.7	− 41.0 ± 6.1	− 391.4 ± 45.7	1505.4 ± 129.6
α11-I domain-(GPP)_2_GPKGER(GPP)_3_	− 60.3 ± 3.4	−7.1	− 30.1.99 ± 4.8	− 290.0 ± 37.0	1109.4 ± 109.2

**Table 2:** Residues contributing to hydrogen bonds between the α11-I domain and triple-helical peptide (GPP)_2_ GFQGEK(GPP)_3_ (chains B, C, and D) is shown. The atom names and the interacting distance (in Angstroms) are shown in square brackets

α11	(GPP)_2GFQGEK_(GPP)_3_		
	Chain B	Chain C	Chain D
TYR139[O]	GLN9[NE2] [2.84]		
ASN141[O]	LYS12[NZ] [2.91]		
ASN141[OD1]	LYS12[NZ] [3.05]		
ASN10[OD1]		GLN9[NE2] [3.03]	
THR77[OG1]		LYS12[NZ] [2.91]	
ASP108[OD2]*		LYS12[NZ]* [2.67]	
GLU110[O]		LYS12[NZ] [2.76]	
ASN14[N]		GLU11[OE2] [3.06]	
ARG142[NE]		GLU11[OE2] [2.63]	
ARG142[NE]*		GLU11[OE1]* [2.63]	
ASN10[ND2]			GLU11[OE2] [2.78]
THR75[OG1]			GLU11[OE1] [2.62]
GLU76[OE1]*			LYS12[NZ] [2.69]*
GLU76[OE2]			LYS12[NZ] [2.69]
HIS112[NE2]			LYS12[O] [2.79]

*Involved in salt bridge formation

The interaction between the α11-I domain and (GPP)_2_GPKGER(GPP)_3_ was distinct compared to the (GPP)_2_GFQGEK(GPP)_3_ interaction with the α11-I domain. Here, much of the interactions come from the Lys9 residue of the strand C collagen peptide (Fig. 7b). Further, Glu76 of the α11-I domain and Lys9 and Arg12 of the collagen peptide from the strand C participated in the H-bond and salt bridge formation. Another salt bridge is formed between Glu110 of α11-I domain and Lys9 of the strand B. Table 3 shows the detailed list of hydrogen bonds and salt-bridges between the acceptor and donor atoms of the α11-(GPP)_2_GPKGER(GPP)_3_ complex.

**Table 3:** Residues contributing to hydrogen bonds between the α11-I domain and triple-helical peptide (GPP)2GPKGER(GPP)3 (chains B and C) is shown. The atom names and the interacting distance (in Angstroms) are shown in square brackets

α11	(GPP)_2_GPKGER(GPP)_3_	
	Chain B	Chain C
GLU110[OE1]	LYS9[NZ] [2.66]	
GLU110[OE2]*	LYS9[NZ]* [2.66]	
TYR140[OH]	PRO6[O] [3.06]	
ASN10[ND2]		PRO6[O] [2.88]
GLU45[OE2]*		LYS9[NZ]* [2.68]
THR75[O]		LYS9[N] [2.75]
THR75[OG1]		GLY7[O] [2.77]
GLU76[OE1]*		LYS9[NZ]* [2.67]
GLU76[OE2]		ARG12[NH1] [3.34]
GLU76[OE2]*		ARG12[NH2]* [2.75]
HIS112[NE2]		LYS9[O] [2.67]

*Involved in salt bridge formation

### Collagen XIII-integrin α11 interaction regulates bone homeostasis in vivo

**Fig. 8:**
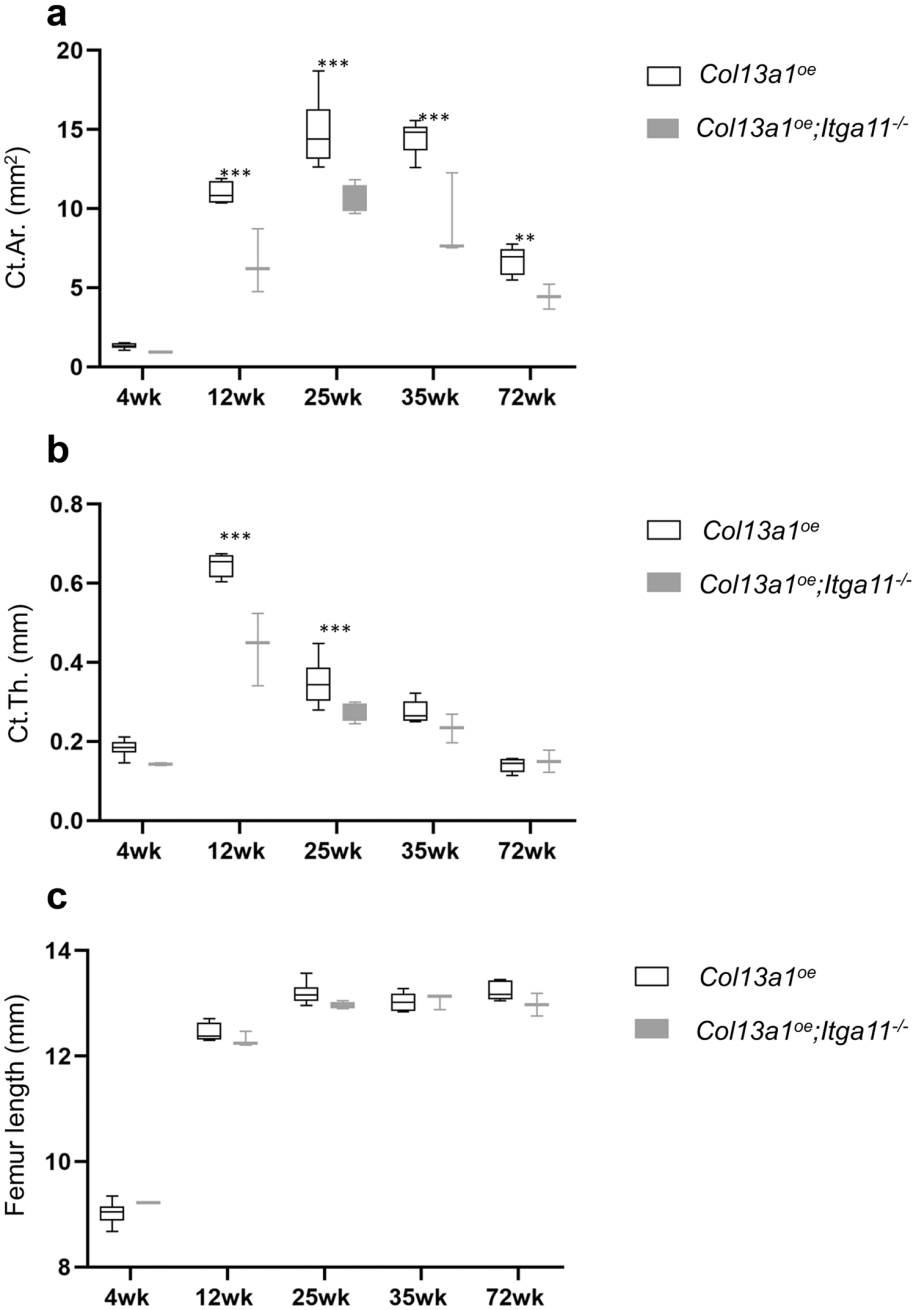
Collagen XIII-integrin α11 interaction affects bone homeostasis. µCT results of cortical bone area (Ct.Ar.) **a** and cortical thickness (Ct.Th.) **b**. No difference was seen in the length of the femurs **c**. Number of mice analyzed in order of increasing age: female *Col13a1*^*oe*^ (7, 4, 6, 6, 6), female *Col13a1*^*oe*^*;Itga11*^*−/−*^ (3, 3, 4, 3, 2). **q < 0.01 and ***q < 0.001 determined by two-way ANOVA followed by FDR

To study the in vivo significance of collagen XIII-α11β1 interaction, we crossbred integrin α11 null mice (*Itga11*^*−/−*^) with mice overexpressing *Col13a1* (*Col13a1*^*oe*^ (Ylönen et al. 2005, Koivunen et al. 2019)) and created a double mutant mouse line (*Col13a1*^*oe*^*;Itga11*^*−/−*^). The *Col13a1*^*oe*^ mice possess a severe bone phenotype, in which especially the circumference of middiaphyseal femur is enlarged (Koivunen et al. 2019). The femoral cortical bone morphology of *Col13a1*^*oe*^ and *Col13a1*^*oe*^*;Itga11*^*−/−*^ mice was evaluated in females at five time points and males at four time points by micro-computed tomography (µCT). As a result, we discovered that the *Col13a1*^*oe*^*;Itga11*^*−/−*^ double mutant mice had a significantly milder bone phenotype when compared to *Col13a1*^*oe*^ mice, the *Col13a1*^*oe*^*;Itga11*^*−/−*^ mice had less cortical bone overgrowth (females in Fig. 8a and males in Fig. S5a) and the bone loss (females in Fig. 8b and males in Fig. S5b) was significantly diminished when compared to *Col13a1*^*oe*^. No differences were observed in the bone length between these genotypes (females in Fig. 8c and males in Fig. S5c). These results indicate that collagen XIII-integrin α11β1 interaction has a role in cortical bone homeostasis, and this role is evident in both genders.

## Discussion

Collagens are known to be recognized by the α-I domains in collagen receptor integrins α1β1, α2β1, α10β1, and α11β1 (Gullberg and Lundgren-Akerlund 2002; Zeltz and Gullberg 2016), and it has been shown that α1β1 and α10β1 interact preferentially with the basement membrane collagen IV and the beaded filament forming collagen VI (Tulla et al. 2001; Nykvist et al. 2000), while α11β1 shows similar features as α2β1 and selects the interstitial collagens (W. M. Zhang et al. 2003). However, collagen IX, a cartilage matrix component, shows exceptionally high binding capacity to all four integrin I domains, suggesting a more complicated mechanism for cell adhesion to collagens (Käpylä et al. 2004). Here, we show the firm interaction of collagen XIII with integrin α11β1.

The motif GFOGER in a triple-helical context has been identified as a target sequence for α1β1, α2β1, α10β1, and α11β1 integrins (Knight et al. 1998, Xu et al. 2000, W. M. Zhang et al. 2003, Sipilä et al. 2014), but these integrins also bind to other ECM proteins that do not contain this particular sequence (Pandolfi et al. 2017). On the other hand, collagen IV does not bind to α2β1 or α11β1 as strongly as collagens I–III, although it contains the GFOGER sequence in the α1(IV), α3(IV), α4(IV), and α5(IV) chains. Additionally, collagen IX, which binds avidly to all integrin I domains, lacks the GFOGER motif. Hence, it has been proposed that GFOGER is not the only target sequence for collagen-binding integrins. Indeed, studies on the integrin-recognition motifs in collagen III have identified GROGER, GLKGEN, and GLOGEN as integrin-binding sites (Raynal et al. 2006; Kim et al. 2005). Moreover, a prokaryotic collagen sequence GLPGER has been reported to bind to integrins α2β1 and α11β1 (Caswell et al. 2008). A study using the collagen peptide ToolKits, which consist of sets of overlapping triple-helical peptides, identified two motifs GLOGEN and GVOGEA that are specific for α1β1 (Hamaia et al. 2012). Additional binding motifs identified for collagen-binding integrins are GFKGER, GLQGER, GLOGER, and GASGER (Zwolanek et al. 2014; Xu et al. 2000).

Our previous study has shown that the ectodomain of the transmembrane collagen XIII binds avidly to the α1-I domain (Nykvist et al. 2000). Here, we show that C2C12 cells expressing α11β1 spread on a collagen XIII-coated surface in the same manner as on a collagen I-coated surface. Among the integrin α1-, α2-, and α11-I domains, the α11-I domain showed strongest binding to collagen XIII, and the collagen XIII showed more specific interaction with the α11-I domain when compared to other collagen types. These data suggest differences in the interaction of collagen XIII with α11 integrin compared to other α subunits.

Collagen XIII is reported to be a ligand mediating integrin α1β1-dependent transmigration of monocytes in Alport renal fibrosis (Dennis et al. 2010). In that study, α1β1-positive monocytes were shown to bind to a GEKGAEGSPGLP sequence located in the COL2 domain of the collagen XIII. In the present study, the α11β1 binding site in collagen XIII was localized in the COL3 domain. Some of the known integrin-binding collagenous motifs, such as GFOGER, GLOGER, and GVPGEA (see above), do not exist in collagen XIII. Furthermore, we did not consider G*XX*GEN-like sequences (*X* = any of the amino acid residues) since these motifs were not preferred in a previous study on the α11-I domain utilizing the collagen peptide ToolKits (W. M. Zhang et al. 2003). In the present study, four motifs, GPKGER, GFQGEK, GSQGEK, and GNRGER from the COL3 domain, and GFPGER, used as a positive control, were chosen for the binding tests. GNRGER showed no binding to the α11, a finding consistent with the ToolKit data. GPKGER, GFQGEK, and GSQGEK bound to α11, but the interaction was more modest than that with GFPGER. Proline hydroxylation is known to be important in collagen-integrin interaction (Sipilä et al. 2018; Rappu et al. 2019). However, in this study, coexpression of prolyl 4-hydroxylase (P4H) in the insect cells did not catalyze the proline hydroxylation probably due to the short (GPP)5 sequence, which was not sufficient for binding of the enzyme P4H to the collagen substrate. Thus, here all prolines are non-hydroxylated. While many previously reported integrin-binding motifs contain hydroxyproline (Rappu et al. 2019), there are also several exceptions, such as GLSGER and GQRGER (Siljander et al. 2004), GFKGER (Zwolanek et al. 2014), GLQGER (Zwolanek et al. 2014), and GASGER (Xu et al. 2000). Moreover, in the hydroxyproline containing functional domains hydroxylation of proline residues is not absolutely required for integrin binding, but it increases the avidity of the interaction. For example, the α2-I domain can bind to GFPGER containing peptides, but more weakly than to GFOGER (Knight et al. 2000).

To model the interaction between the α11-I domain and collagen motifs, two different collagen peptides ((GPP)_2_GFQGEK(GPP)_3_ and (GPP)_2_GPKGER(GPP)_3_ were constructed through in silico approaches. The docking analysis showed that both constructed triple-helical peptides indeed bind to the interface of α11-I domain. In the crystal structure of the integrin α2-I domain bound to the triple-helical GFOGER peptide (J. Emsley et al. 2000), the strand C makes most of the interactions with the α2-I domain whereas the strand B contributes fewer interactions and the strand D makes no interactions as it is exposed to the solvent. Consistently, this trend was observed in our docking experiments for the GPKGER peptide, where most of the hydrogen bonds came from the strand C, less hydrogen bonds from the strand B and no interaction from the strand D. It can be hypothesized that the presence of an arginine (Arg12, the 12th residue in the strand C) residue might influence the orientation of the triple-helical peptide with the integrin domain such that the strand C makes many contacts with the integrin I domain similar as seen in the template crystal structure. However, for the GFQGEK, most of the hydrogen bonds came from the strand C and multiple hydrogen bonds were observed from both the strands B and the D. It is conceivable that the change of arginine (Arg12) to lysine (Lys12) may disrupt the orientation exposing all the three strands contacting the I domain. Consequently, our experimental data supports the molecular modeling results as C2C12-α11+ cells attach much stronger on GPKGER than on GFQGEK (Fig. 4d). Homology modeling and docking can be considered a powerful strategy to predict protein-protein interactions if there is no crystal structure of the complexes available (Quignot et al. 2018). Accuracy of the docking depends on the quality of the homology model (Quignot et al. 2018; Rodrigues et al. 2013); thus, we have validated our model at different stages both manually as well as using various online servers to obtain a reliable prediction of the α11-I domain structure and the docked complex (see “Materials and methods”). However, despite striving for high-quality predictions in our study, it should be noted that the crystal structures of the α11-I domain in an unbound and a collagen-bound state remain to be solved, allowing further confirmation of our modeling results.

The peptide sequence GPKGER exists in 15 different collagens (collagens III, IV, V, VII, IX, XI, XIII, XIV, XV, XVII, XIX, XX, XXII, XXV, XXVII), while GFQGEK exists only in collagens XIII and IV, and GSQGER only in mouse collagen XIII and in rat XVII (UniProt Consortium 2019). It is not clear why collagen IV does not bind to α11 although it contains both GFOGER and GFQGEK sequences (Farndale et al. 2008, W. M. Zhang et al. 2003). Collagen IX, which binds to all the four I domains, contains the GFKGER sequence also. We propose GFKGER as a new general integrin-binding motif, albeit with modest affinity.

In the light of data obtained with cells and mutant mice (Hägg et al. 1998, 2001; Kvist et al. 2001; Sund et al. 2001; Tahkola et al. 2008; Tuomisto et al. 2008), collagen XIII appears to play a role in cell-matrix adhesion. Recent in vivo studies highlight involvement of collagen XIII in the development, differentiation, and maturation of musculoskeletal tissues and vessels and in maintaining tissue integrity (Latvanlehto et al. 2010; Härönen et al. 2017; Zainul et al. 2018; Heikkinen et al. 2019; Koivunen et al. 2019). The 150-nm flexible ectodomain of collagen XIII can potentially cross the basement membrane around myofibrils or under endothelium and can penetrate into the surrounding stroma. Thus, the in vitro properties of collagen XIII in binding to the basement membrane collagen receptor α1β1 and to the matrix interstitial collagen receptor α11β1 may be of physiological relevance. Moreover, COL3, the furthest collagenous domain from the plasma membrane, appeared largely responsible for the binding to the α11-I domain, suggesting that collagen XIII molecules could interact with integrin α11β1 molecules occurring in neighboring cells, but this aspect was not studied in the present study.

Under physiological conditions, integrin α11β1 is expressed in specific subsets of fibroblasts and mesenchymal stem cells (MSCs) (Popov et al. 2011; Shen et al. 2019) and it has previously been observed to be up-regulated in carcinoma-associated fibroblasts (CAFs) in the tumor stroma (Zhu et al. 2007; Smeland et al. 2019). Collagen XIII is shown to be up-regulated by TGF-β in the reactive stromal cells of epithelial tumors and throughout mesenchymal tumors. The induction of collagen XIII expression occurs at an early stage in tumor progression in response to malignant transformation (Väisänen et al. 2005).

Integrin α11β1 is a regulator of MSC survival on collagen I (Popov et al. 2011). Moreover, α11β1 is found in leptin receptor positive MSCs in the bone marrow where α11β1 has shown to be an osteolectin receptor and a regulator of osteogenesis and bone homeostasis (Shen et al. 2019). Conditional deletion of the α11 subunit from leptin receptor positive bone marrow cells accelerates trabecular bone loss in aged mice without alterations to body size, femoral length or cortical bone area. However, the mineral apposition rate is significantly reduced in cortical bone in these mice compared to littermate controls. We have previously shown that collagen XIII regulates bone remodeling and angiogenesis through β1 integrins (Koivunen et al. 2019). The novel double mutant mouse line presented here, *Col13a1*^*oe*^*;Itga11*^*−/−*^, has a milder high bone mass phenotype compared to *Col13a1*^*oe*^ mice. More specifically, double mutants have significantly less bone overgrowth and milder osteoporosis compared to *Col13a1*^*oe*^ mice. A comparable phenotype was seen in both sexes up to 35 weeks of age. We were not, however, able to complete a full cohort of the male mice to the final time point on account of insufficient amount of mice available for the experiment. These data suggest that collagen XIII-triggered effects in bone homeostasis may be mediated by α11β1. However, *Itga11* deficiency does not lead to full rescue of the bone phenotype indicating existence of other, unknown, mechanisms triggered by collagen XIII.

In conclusion, we identify integrin α11 as a new collagen XIII receptor and show that this ligand/receptor pair may have a role in bone homeostasis. Additionally, we propose GFKGER as a new general integrin-binding motif with moderate affinity.

## Electronic supplementary material

Below is the link to the electronic supplementary material.

Supplementary file1 (PDF 13.6 MB)
