# Supplementary material for: Integrin α11β1 is a receptor for collagen XIII
Source: Cell Tissue Res. 2020 Dec 11;383(3):1135–53. doi: 10.1007/s00441-020-03300-y (PMC7960628; doi:10.1007/s00441-020-03300-y)
Supplement: Supplementary file 1 — Supplementary file1 (PDF 13.6 MB) [file 441_2020_3300_MOESM1_ESM.pdf]

sFigure 1.

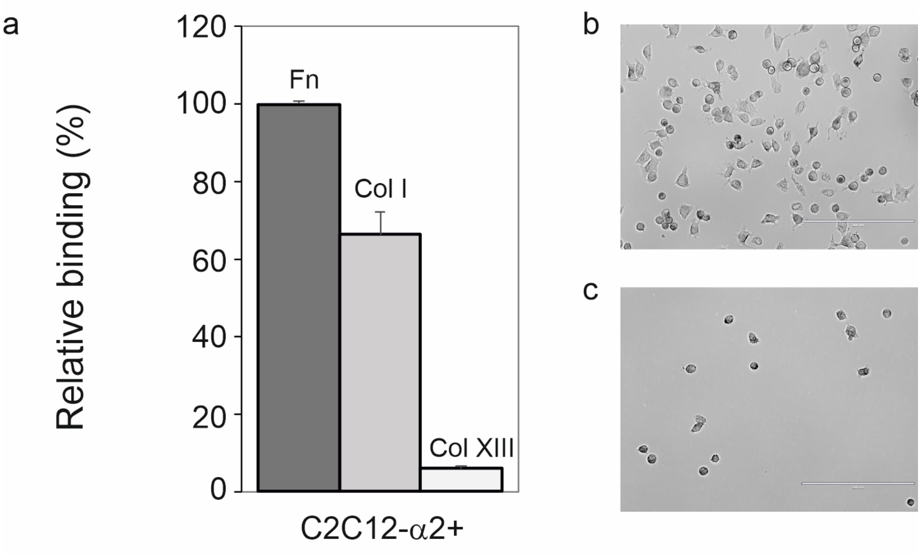

sFigure 1. Adhesion of integrin α2-transfected C2C12 cells to fibronectin and collagens (a) C2C12 control cells and cells transfected with an integrin α2cDNA (C2C12-α2+) were seeded on a plastic surface coated with fibronectin, collagen I or the collagen XIII ectodomain. The cells were analyzed quantitatively using the β-hexosaminidase release assay for the attachment to fibronectin, collagen I, and collagen XIII. Data are presented relative to fibronectin and BSA binding, where the saturated binding to fibronectin represents 100 % and the background binding to a BSA-coated surface represents 0 %. Values are shown as means of triplicates with standard deviation bars. Microscopic monitoring of the attachment and morphology of C2C12-α2+ cells adhering on collagen I (b) and collagen XIII (c). Scale bars 200 µm.

sFigure 2.

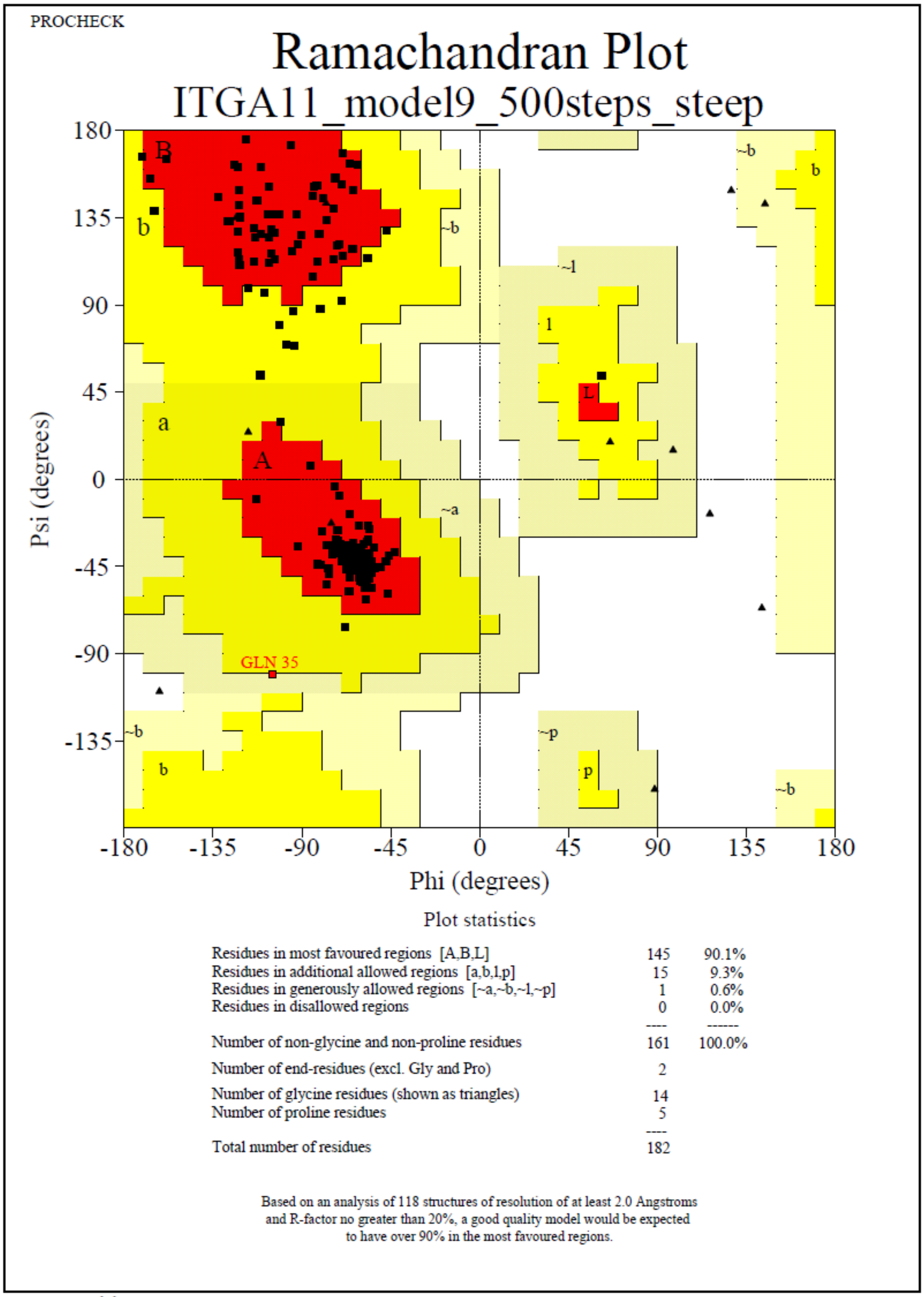

sFigure 2. Ramachandran plot of the energy minimized structure of the modeled human integrin α11-I domain shows 90.1% of the amino acid residues are in most favoured regions while 9.3% and 0.6% of the residues are in the additional allowed and generously allowed regions respectively. None of the residues are in disallowed regions.

sFigure 3.

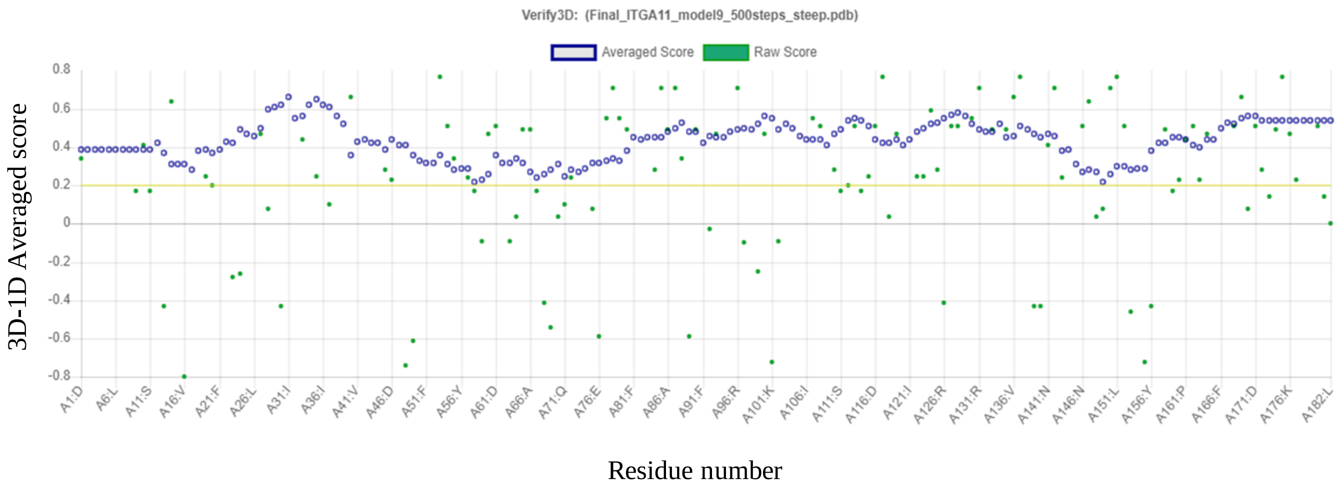

sFigure 3. Verify 3D prediction. The Verify 3D predicted that 100% of the residues in the α11-I domain had an average 3D-1D score > 0.2, suggesting that the constructed model is of high quality and more reliable.

sFigure 4.

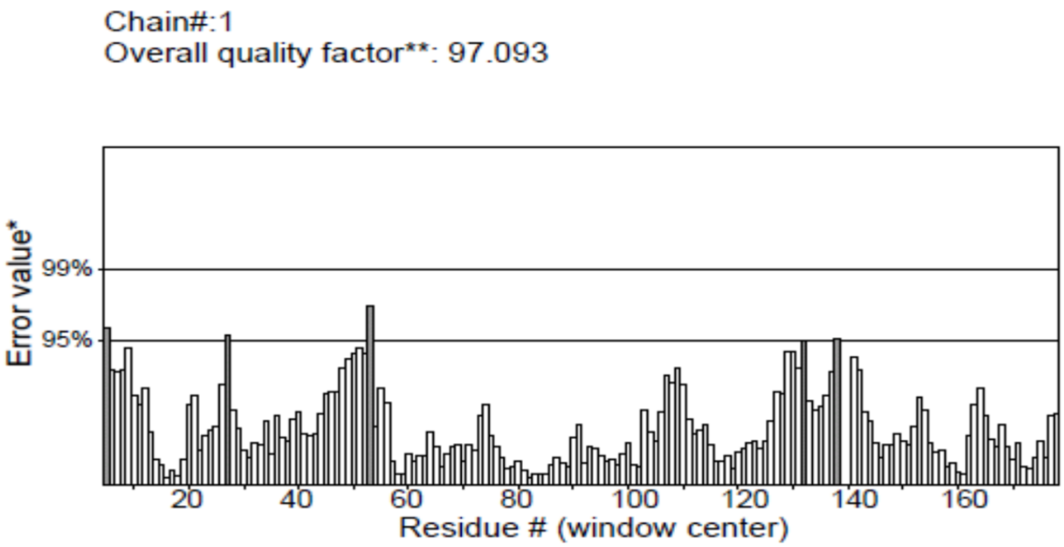

sFigure 4. Errat plot for the α11-I domain model. The gray bars are the error region between 95% and 99%, and the white bars denotes the region with lower error rate. In general, the protein structure with the quality range of >50 is considered as a high-quality model. For the constructed α11-I domain model, the overall quality factor predicted by the ERRAT server was 97.093.

sFigure 5.

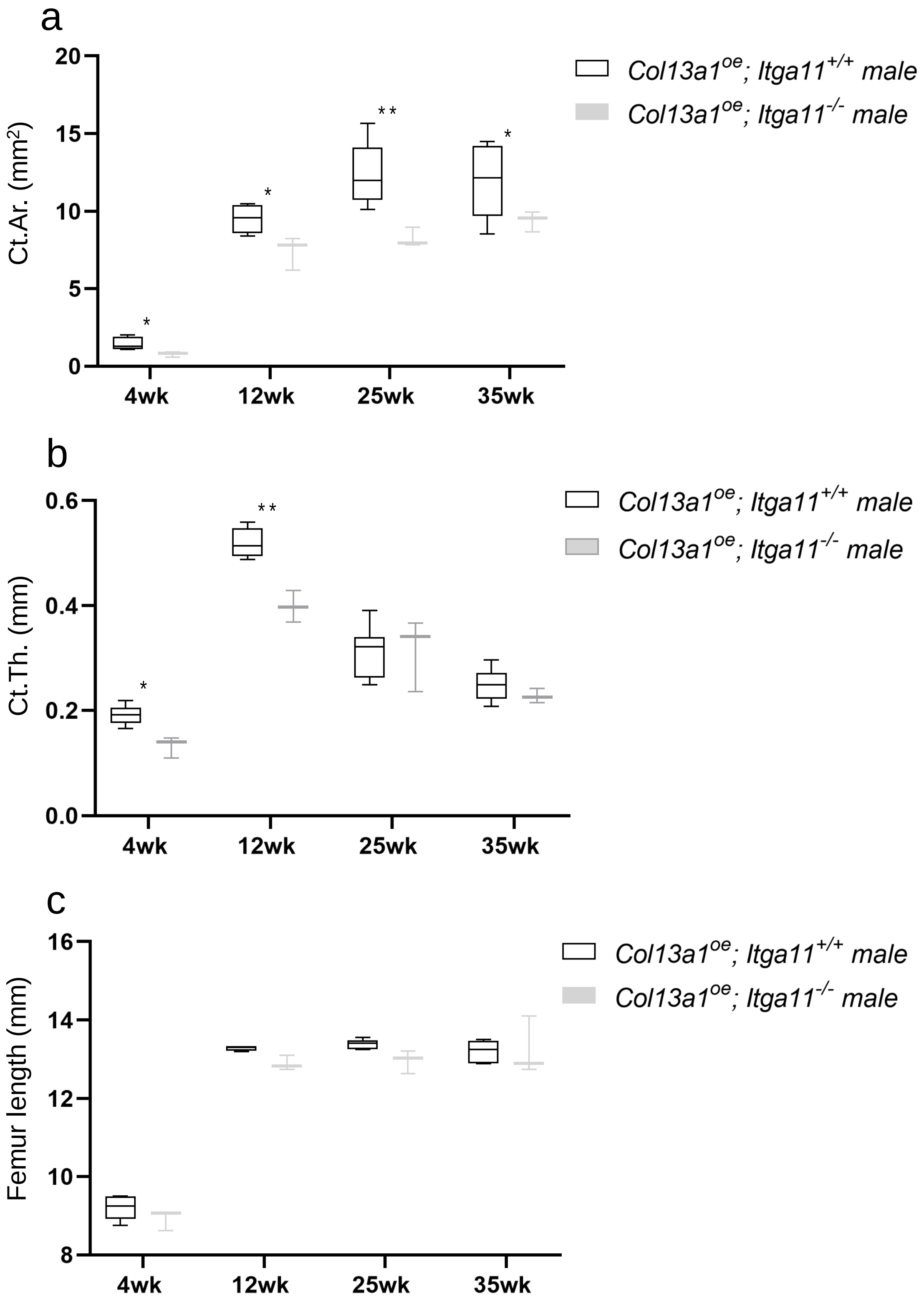

sFigure 5. Collagen XIII-integrin α11 interaction affects bone homeostasis in male mice. µCT results of cortical bone area (Ct.Ar.) (a) and cortical thickness (Ct.Th.) (b). No difference was seen in the length of the femurs (c). Number of mice analyzed in order of increasing age: male *Col13a1*^{oe} (5, 4, 7, 6), male *Col13a1*^{oe};*Itga11*$^{-/-}$ (3, 3, 3, 3). *$q < 0.05$, and **$q < 0.01$ determined by two-way ANOVA followed by FDR.
